# “A breach in the protocol for no good reason”: a surgical antimicrobial prophylaxis experience in an Ethiopian academic medical center

**DOI:** 10.1186/s13741-023-00328-w

**Published:** 2023-07-13

**Authors:** Veronica Afework, Segni Kejela, Nebyou Seyoum Abebe

**Affiliations:** Department of Surgery, College of Health Sciences, Addis Ababa University, Addis Ababa, Ethiopia

**Keywords:** Surgical antimicrobial prophylaxis, Surgical site infections, Gastrointestinal surgery

## Abstract

**Background:**

An appropriately administered surgical antimicrobial prophylaxis decreases the rate of surgical site infections. Although evidence-based clinical practice guidelines have been published on surgical antimicrobial prophylaxis, the rate of adherence to the protocol and the impact of extending antimicrobial prophylaxis postoperatively is yet to be well elucidated.

**Method:**

A total of general surgery and vascular surgery patients with clean and clean contaminated wound undergoing elective surgical procedures were included in the study. The rate of surgical antimicrobial prophylaxis utilization, the proportion of patients whom had their antimicrobial prophylaxis extended beyond 24 h and the rate of surgical site infections across groups were evaluated.

**Results:**

The surgical antimicrobial prophylaxis utilization rate was 90.5%. Of these patients, 12.6% were unnecessarily administered with antibiotics. An “extended” antibiotics administration beyond 24 h after the surgery was found in 40.2%. Gastrointestinal and hepato-pancreatico-biliary surgery patients had 7.9-fold rate of “extended” surgical antimicrobial prophylaxis beyond 24 h, AOR 7.89 (95% CI 3.88–20.715.62, *p* value < 0.0001). The overall rate of surgical site infection was 15(6.8%). The “extended” regimen of prophylactic antibiotics had no effect on the rate of surgical site infections.

**Conclusion:**

Less than half of the patients included here had surgical antimicrobial prophylaxis regimen in accordance with the existing guidelines. The most common protocol violation was noted as extension of antimicrobial prophylaxis for more than 24 h after surgery. The extension of antimicrobial prophylaxis did not decrease the rate of surgical site infections, reaffirming the evidence that prophylactic extension of surgical antimicrobial prophylaxis is unnecessary.

## Introduction

Surgical site infections (SSI) are the most common nosocomial infections (Mangram et al. 1999). An appropriately administered surgical antimicrobial prophylaxis (SAP) decreases the rate of SSI by half (Geroulanos et al. 2001). SAP protocols are put in place because SSIs are responsible for increase in length of hospital stay, re-admissions, cost and mortality (Zimlichman et al. 2013; Broex et al. 2009).

It is found that there is poor adherence to SAP protocols on institutional and national levels in most Asian and African countries (Afzal Khan et al. 2013; Gul et al. 2005; Sandt et al. 2019). This poor adherence to the protocols has been attributed to lack of awareness of the existence of the protocols, reliance on old habits and skills learned during medical school and residences, and values and beliefs strongly held by the practitioners despite no evidence to support it (Hassan et al. 2021).

In the Ethiopian context, few studies have shown a variable patterns of antibiotics prophylaxis in the perioperative period. But determination of the appropriateness of the use and adherence to the existing guidelines and recommendations is yet to be explicated (Alamrew et al. 2019). Here we aim to determine the rate of SAP utilization, the appropriateness of the existing pattern of antibiotic use in the perioperative period, the rate of “extended” prophylactic administration of antibiotics beyond the time limit for SAP protocols and whether there is any impact of the “extended” use on the rate of surgical site infections compared to the standard use.

## Methods

### Study design

We conducted an institutional retrospective study on patients that underwent elective open surgical interventions over the course of a 3-year period, from June 2018 to June 2021. The patients involved in the study were treated under the surgical units of breast and endocrine surgery, hepato-pancreatico-biliary surgery, colorectal surgery and vascular surgery.

### Study setting

This study was conducted at Tikur Anbessa Specialized Hospital, the largest academic medical center in Ethiopia. It is located in the capital city, Addis Ababa and provides both medical and educational services at a sub-specialty level.

### Sample size

A 50% population proportion was used because of absence of previous studies which brought the sample size to 384. Only 244 patients during the time period fulfilled the inclusion criteria. Using the finite population calculation the sample size became 150. But to increase the power of the study and improve on its generalizability, all the patients that fulfilled the inclusion criteria were included.

### Study participants

All patients with age of ≥ 14 years whom underwent major surgical intervention using open technique with clean and clean contaminated wounds over the 3 years period of the study.

### Inclusion criteria

All elective surgery patients that underwent open surgery for endocrine surgery, breast surgery, hepato-pancreato-biliary surgery, colorectal surgery, and upper GI surgery are included. All included patients had clean and clean contaminated wound class. Every patient that were include are of the age of ≥ 14 years.

### Exclusion criteria

All pediatric patients that were surgically managed at the hospital.

Patients admitted for nonsurgical or minimally invasive modes of treatment and those that were either discharged or died before any surgical intervention was performed.

### Study variables

#### Independent variables

Age, gender, comorbidities, wound classification, type of surgery, prophylactic antibiotics administration, timing of antibiotics administration, intraoperative blood loss, duration of surgery, and re-administration of antibiotics.

#### Dependent variables

The primary outcome was the rate of “extended” postoperative antibiotics administration, and the secondary outcome was the rate of surgical site infections.

### Data sources

The data were collected from the patients’ medical records retrieved physically from a paper chart using a data collection tool created and validated before the start of the study. This tool included socio-demographic, clinical, and outcome data.

### Measurement/analysis and interpretation

After the data have been collected, it was coded, cleaned, and entered into IBM SPSS Statistics for Windows, Version 24.0. Armonk, NY: IBM Corp. The outliers were sought for and corrected. Afterward, the data was analyzed using the same software using a descriptive and inferential statistical models.

### Study size

All patients fulfilling the inclusion criteria over the predetermined study period of 3 years well included in the study.

### Statistical methods

Initially, descriptive analysis was used to determine the mean and standard deviations of the parametric variables used in the study. Categorical variables were analyzed using frequency distribution tables. Dependent variables were cross-tabulated with the independent study variables, and univariable analysis was performed. Those factors that were determined by the univariable analysis to potentially impart the dependent variables’ outcomes were analyzed with multivariable logistic regression and the values were presented as adjusted odds ratios with a p-value.

### Ethical considerations

Ethical approval was acquired from the Institutional Review Board of Addis Ababa University, College of Health Sciences, which is the governing body of health sciences researches under the jurisdiction of Addis Ababa University. The study was conducted in accordance to Helsinki declarations, Ethiopian National Research Ethics Guidelines, and Institutional regulations on research ethics. All the data collected were handled confidentially and no entity outside of the investigators had any access to any of the patient information.

## Results

**Fig. 1:**
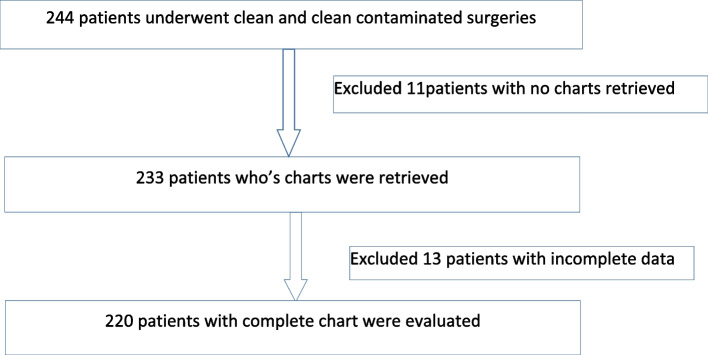
Flow chart of included and excluded patients with missing chart and incomplete data content

A total of 244 patients underwent clean and clean contaminated wound class surgery, of which 220 were evaluated while the rest were excluded because of missing charts and incomplete information (Fig. 1). Of the total of 220 patients included in the study, 114 (51.8%) were females. The mean age was 43.9 ± 15.4 years with the age range of 14–90 years. Clean and clean contaminated wounds were 48.2% and 51.8% respectively. The mean duration of surgery was 2.52 ± 1.43 h with a range of 0.2 to 7.5 h. Participant demographics are shown in Table 1. Hepato-pancreatico-biliary surgeries and vascular surgeries had the highest number of surgical interventions performed, and 143(65%) of the patients had blood loss of less than 500 ml. The number of surgical interventions performed in each units, amount of blood loss, and the duration of the surgeries are shown in Table 2.

**Table 1:** Socio-demographic factors of the patients

**Variables**	**Category**	**Number**	**Percentage (%)**
**Gender**	Male	106	48.2
	Female	114	51.2
**Comorbidities**	Diabetes	6	2.7
	Hypertension	17	7.7
	Retroviral infection	7	3.2
	Diabetes + hypertension	10	4.5
	Other	17	7.7
	None	163	74.1
**Class of wound**	Clean wound	106	48.2
	Clean-contaminated wound	114	51.8

**Table 2:** The type of surgery performed, the estimated blood loss and duration of surgery

**Category**	**Variables**	**Number**	**Percentage (%)**
**Type of surgery**	Thyroid surgery	18	8.2
	Breast surgery	13	5.9
	Hernia surgery	2	0.9
	Upper GI surgery	15	6.8
	Hepato-pancreatico-biliary surgery	65	29.5
	Colorectal surgery	33	15
	Skin and deep tissue surgery	6	2.7
	Vascular surgery	60	27.3
	Splenectomy/adrenalectomy	8	3.6
**Estimated blood loss**	Less than 500 ml	143	65
	500–999 ml	41	18.6
	1000–1499 ml	11	5
	1500 ml or more	10	4.5
	Unreported	15	6.8
**Duration of surgery**	< 4 h	179	81.4
	4 or more hours	41	18.6

**Fig. 2:**
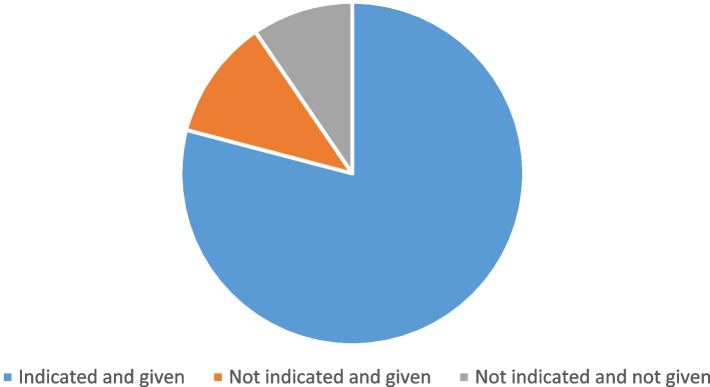
Proportion of patients with indicated antibiotics and administered, not indicated but administered, and not indicated not administered

A total of 199(90.55%) of the patients received prophylactic perioperative antibiotics of any kind, of which 174(87.4%) had a clear indication for the administration (Fig. 2). The majority, 118(53.6%) of the patients had antibiotic administration at the time of induction. Of those administered with antibiotics preoperatively 40.2% had antibiotics extended beyond 24 h after surgery. All patients were administered with Ceftriaxone 1 gm IV only (Table 3).

**Table 3:** Duration of postoperative antibiotics administration

**Category**	**Variables**	**Number**	**Percentage (%)**
**Timing of prophylactic antibiotics**	At the time of induction	118	59.3
	Less than 30 min before induction	12	6.0
	30–60 min before induction	38	19.1
	1–2 h before induction	3	1.5
	Unknown	28	14.1
Duration of antibiotic administration	No postoperative administration	44	22.1
	Less than 24 h	75	37.7
	24–48 h	28	14.1
	48–72	12	6.0
	> 72 h	40	20.1

“Extended” antibiotics administration beyond 24 h after surgery was found in 63(55.8%) of the 113 patients undergoing gastrointestinal and hepato-pancreatico-biliary surgeries, compared to only 17(15.9%) of the 107 patients undergoing soft tissue, endocrine, vascular, splenic, and hernia surgeries. On multivariable analysis, gastrointestinal and hepato-pancreatico-biliary surgery patients had more than 7.9-fold more likelihood of having antibiotics administered for more than 24 h after surgery compared to the other surgical procedures, adjusted odds ratio 7.89 (95%CI 3.88–20.715.62, *p* value < 0.0001). None of the other variables including duration of surgery, volume of intraoperative blood loss, class of wound, age, and comorbidities affected the duration of postoperative antibiotics administration.

The overall rate of surgical site infection (SSI) was 15(6.8%).The rate of SSI in clean and clean contaminated wound was 4.7% and 8.8% respectively. The rates of SSI among patients with SAP administered for less than 24 h and more than 24 h were 4.3% and 11.3% respectively. The type of surgery performed, the duration of perioperative antibiotics, the class of wound and the length of the procedure had no association to the rate of SSI on a multivariable analysis.

## Discussion

In this study involving elective surgical patients with a clean and clean contaminated wounds violation of the standard guidelines was found in just more than half of the patients included, with 12% unnecessary initial administration and 40% unnecessary extension of the antimicrobials. Furthermore, abdominal surgery is shown to be the only factor associated with “extended” antimicrobial prophylaxis beyond 24 h. Despite this, the rate of SSI was not decreased by the “extended” administration disproving the need to ever administer prophylactic antibiotic beyond 24 h after the surgery.

Historically perioperative antimicrobial type, mode and duration of administration were left to the discretion of the surgeon as there were no guiding principles (Westerman 1984). This helter-skelter fashion of SAP was gradually replaced by multitude of constantly updating guidelines (Westerman 1984). The unifying themes of these guidelines are narrow spectrum of antibiotics choice, administration within an hour of incision and limiting SAP duration to less than 24 h of the surgery (Bratzler et al. 2013; Scottish Intercollegiate Guidelines Network 2008; Preventing surgical site infections 2012; High impact intervention; care bundle to prevent surgical site infection 2011). These themes should guide every SAP related practices in all institutions since they are supported by surplus of evidences (Weber et al. 2017; Nagata et al. 2022; Meijer et al. 1990; Ahmed et al. 2022). Nonetheless adherence to SAP protocols in the low and middle income countries is below 50% with the most common failure in adherence being an unnecessarily extended postoperative antimicrobial administration (Satti et al. 2019; Abdel-Aziz et al. 2013; Alahmadi et al. 2020). These figures are consistent with our findings.

Historically, patients undergoing abdominal surgeries were subjected to an “extended” antibiotics course after surgery before the introduction of the guidelines. This patient population included hepatectomy surgeries, colectomy, gastrectomy, small bowel surgery, and biliary interventions (Kappstein and Daschner 1991; Murtha-Lemekhova et al. 2022). Similar group of patients in our series also were subjected to more days on antibiotics beyond the recommended time for SAP. Surgeons’ preferences and biases have led to such discrepancies in duration of antibiotic administrations across different surgeries in the abdomen (Aoun et al. 2005).

The rate of SSI in our patient cohort was lower than the 25% reported from pooled analysis Ethiopian population in a systematic review (Birhanu and Endalamaw 2020). But few of the studies included in the systematic review had prevalence rates closer to our findings than the pooled value iterating an institutional influence in the rate of SSI (Birhanu and Endalamaw 2020; Gelaw et al. 2014, 2017).

Studies had failed to provide any indication for an “extended” SAP utilization. For instance, in patient population undergoing elective hepatectomy, “extended” antibiotic administration had no impact on the rate of SSI, but increased the number of patients with methicillin resistant staphylococcal infections (Kappstein and Daschner 1991). Similarly, in those undergoing gastrectomy the prolonged use group had no reduction in the rate of SSI detection (Lee et al. 2010). Whether it is benevolence for an individual patient, fear of the unknown or assumption that it decreases the rate of SSI, “extended” SAP should have no place in the modern evidence-based clinical practice (Mmari et al. 2021; Broom et al. 2018).

Patients undergoing colorectal surgery are shown to benefit from administration of metronidazole in addition to cephalosporins with an overall SSI reduction rate of 66% compared to cephalosporin alone (Nelson et al. 2014; Jewesson et al. 1997). Furthermore, updated guidelines support the use of metronidazole for anaerobic coverage based on the high-quality data in existence (Bratzler et al. 2013; Scottish Intercollegiate Guidelines Network 2008; Preventing surgical site infections 2012; High impact intervention; care bundle to prevent surgical site infection 2011). None of the studied patients here undergoing colon and rectal surgery had anaerobic SAP coverage further violating the guidelines.

The limitations of this study includes the its retrospective design and its associated biases. In addition, pediatric and pregnant population have not been included in the study, so it would not be possible to generalize the findings to these groups.

In conclusion, violation of SAP protocols was seen in nearly half of the patients in the group of patients studied here. Abdominal surgery patients had close to 8-fold higher rate of antibiotics extension beyond 24 h postoperatively. Despite the “extended” administration of SAP this group had similar levels of surgical site infections as those without extension of SAP administration, reiterating that a breech in protocol leading to a longer antibiotics utilization has no value in reducing the rate of surgical site infections. Surgeons and institutions should strive to abate the practice of unnecessarily prolonged antibiotics utilization and adhere to the existing guidelines.

## Data Availability

Source data will be available upon reasonable request to the corresponding author.
